# Clinical and magnetic resonance imaging features, and pathological findings of spinal lymphoma in 27 cats

**DOI:** 10.3389/fvets.2022.980414

**Published:** 2022-10-20

**Authors:** Valentina Lorenzo, João Ribeiro, Marco Bernardini, Juan J. Mínguez, Meritxell Moral, Carlos Blanco, Tina Loncarica, Araceli Gamito, Martí Pumarola

**Affiliations:** ^1^Neurología Veterinaria, Getafe, Madrid, Spain; ^2^Referência Veterinária, Cascais, Portugal; ^3^Faculdade de Medicina Veterinária da Universidade Lusófona de Humanidades e Tecnologias de Lisboa, Lisbon, Portugal; ^4^Anicura Ospedale Veterinario I Portoni Rossi, Zola Predosa, Italy; ^5^Department of Animal Medicine, Production and Health, University of Padua, Padua, Italy; ^6^Hospital Veterinario Guadiamar, Sevilla, Spain; ^7^Scarsdale Vets-Pride Veterinary Centre, Derby, United Kingdom; ^8^Mouse and Comparative Pathology Unit, Department of Animal Medicine and Surgery, Veterinary Faculty, Campus Universitat Autónoma de Barcelona (UAB), Barcelona, Spain

**Keywords:** feline, MRI, imaging, neoplasia, spinal cord, CSF, cytology, histopathology

## Abstract

This multicentric retrospective study describes the clinical and MRI features and pathological studies of spinal lymphoma in 27 cats. MRI characteristics and their possible correlations with histopathological findings were studied. The most frequent neurological signs were rapidly progressive paraparesis (62.9%) or paraplegia (22.2%). Bimodal age distribution was found with 40.7% of cats aged ≤2.5 years (63.6% of them FeLV positive), and 44.4% of cats aged ≥8 years (16.7% of them FeLV positive). Spinal lymphoma was generally presented on MRI as an ill-defined epidural focal lesion with moderate to severe spinal cord compression, expanding more than one vertebral body. MRI lesions were typically localized in the lumbar vertebral segment (*p* = 0.01), circumferential to the spinal cord (*p* = 0.04), hyperintense on T2-weighted sequences (*p* = 4.3e-06), and isointense on T1-weighted sequences (*p* = 8.9e-07). The degree and pattern of contrast enhancement were variable. Other morphological patterns included paravertebral masses with extension into the vertebral canal and lesions centered in the spinal nerve roots. Involvement of vertebrae and adjacent spinal soft tissues was present in 74% of cases when present vertebral involvement was characterized by cortical sparing. When follow-up MRI studies (*n* = 4) were performed after treatment new lesions of similar nature but different localizations and extension were observed. Confirmation of spinal lymphoma was performed by CSF analysis in 4/27 (14.8%) of cases, by FNA in 6/27 (22.2%) of cases, by surgical biopsy in 10/27 (37%) of cases, by FNA and surgical biopsy in 1/27 (3.7%) of cases, by CSF, FNA, surgical biopsy and postmorten examination in 1/27 (3.7%) of cases, and postmorten studies in 5/27 (18.5%) of cases. Antemortem diagnosis was achieved in 22/27 (81.5%) cats. The presence of necrosis in histopathological studies as an unfavorable prognostic indicator of survival was significantly more probable when lesions were not hyperintense on T2-weighted sequences (*p* = 0.017). Spinal lymphoma in cats is a complex entity with heterogeneous imaging and histopathological appearance. However, certain MRI features may support a tentative diagnosis, which in a group of cases can be confirmed when combined with the CSF findings. For the rest of the cases, tissue sampling assisted by imaging findings remains necessary for definitive diagnosis.

## Introduction

Lymphoma is the most common spinal neoplasia in cats (1–3) and the second most common disease of the spinal cord (1, 2, 4, 5) but information about Magnetic Resonance Imaging (MRI) features is still scarce. To date, case series of feline spinal lymphoma have been limited to a few reports including limited cases (3, 6–8).

The few descriptions of MRI findings, together with the heterogeneous histopathological nature of this disease (9) hampers precise prognosis and clinical management. MRI, as being the gold standard technique for assessing the nervous system, is highly valuable for prompt diagnosis of neoplasia. In fact, in humans, presumptive antemortem diagnosis of lymphoma affecting the nervous system is based on the identification of MRI-specific morphologic features (10–12), and the correlation of MRI features in primary brain lymphoma with histopathologic findings has been studied (12). Moreover, as MRI allows the characterization of the distribution of the lesions it may facilitate tissue sampling for diagnosis confirmation by cytology or histopathology and therefore facilitate adequate treatment and prognosis evaluation.

The goals of this study were to describe the clinical and MRI characteristics of feline spinal lymphoma in 27 cats and to compare the findings with previous reports in an attempt to identify characteristics that may aid in antemortem diagnosis. Moreover, possible correlations of MRI features with the histopathological findings which may assist prognosis were investigated.

## Materials and methods

Medical records including the clinical data, the MRI studies with their corresponding reports, and the results from the pathology studies of spinal lymphoma affecting the nervous system in cats from 5 neurology referral centers were retrieved by a board-certified or a residency-trained neurologist. MRI studies were reviewed by two authors blinded to the final diagnosis at the time of image evaluation and the final result was achieved by consensus. All the information was retrospectively reviewed by a board-certified neurologist (VL). Cases were included in the study if presumptive diagnosis on spinal cord MRI was confirmed based on evidence of neoplastic cells in the CSF and/or in tumor samples. Cases were not included if the results were inconclusive.

Information regarding age, sex, feline leukemia virus (FeLV) status, neurological evaluation, duration of clinical signs prior to MRI studies, survival time, treatment [medical management -steroids, non-steroidal anti-inflammatory drugs (NSAIDs), antibiotics, analgesic, or some combination of these-, chemotherapy, surgery, or combination of these] and outcome (survival time from the day of MRI) was retrieved.

Cases were categorized by tumor location in relation to the vertebral column on MRI, and together with the clinical data, a presumptive diagnosis was generated. Cases were finally diagnosed and further subclassified into tumor types according to clinicopathological and anatomopathological studies.

The sampling and the imaging procedures were held in a clinical environment as part of the diagnostic protocol for medical purposes, with the signed consent of the owners. The material used for the study did not require supplementary procedures or handling.

### MRI study acquisition and evaluation

MRI studies were performed using 3 different 1.5-Tesla magnets (Gyroscan Intera and Prodiva, Philips, Eindhoven, The Netherlands; Vantage Elan, Canon Medical Systems Europe, Zoetermeer, The Netherlands), a 0.5-Tesla magnet (Gyroscan, T5-II, Philips, Eindhoven, The Netherlands) and 3 different 0.2-Tesla magnets (MrJ, Paramed, Genoa, Italy; Hitachi Airis mate, Chiyoda, Japan; Vet MRI, Esaote, Genoa, Italy).

All MRI studies were performed under inhalation anesthesia with the patient in dorsal recumbency. The area of study of the vertebral column was based on the clinical findings and neurolocalization and extended if necessary according to the findings on each particular case. MRI protocols included at least T2-weighted (T2W) sequences in sagittal and transverse planes and T1-weighted (T1W) sequences before and after intravenous administration of gadolinium-based contrast medium (Gadoteric acid, Dotarem, Guerbet, or Claricyclic, GE Healthcare, at a dose of 0.1 mmol/Kg).

Cases were categorized by tumor location in reference to the spinal cord (intramedullary, intradural extramedullary, epidural, spinal nerve root/s) and located in relation to the corresponding vertebral body/ies. Furthermore, lesions were classified as solitary (focal or extensive) or multifocal. The lesion was considered extensive if it extended over the length of >1 vertebral body considering the vertebral canal component.

The signal intensity on T1W, T2W, and additional STIR sequences was defined as iso- hypo- or hyperintense compared to normal spinal cord parenchyma.

The degree of contrast enhancement when pre- and post-contrast images were compared was subjectively defined as none, mild, moderate, or intense, and the type as homogeneous or heterogeneous. Meningeal contrast enhancement was recorded if present.

Additional recorded features included the presence and degree of spinal cord compression (mild, <25%; moderate, 25–50%; severe, >50% occupation of the vertebral canal diameter) and other possible spinal cord changes.

When the vertebral bone or other adjacent bone structure was involved, changes in the bone marrow pre and post-contrast images, the presence of cortical lysis (discontinuity of the normal cortical margin), cortical sparing (preservation of the normal cortical margin), and preservation of vertebral shape (preservation of the overall architecture of the vertebra despite small foci of cortical lysis) were studied. If present, involvement of soft tissues (paraspinal and/or distant) was registered.

Follow-up MRI studies after surgery and/or chemotherapy were performed on 4 cats. When a second MRI was performed findings on the area of the previous lesion/s and new findings were recorded.

### CSF and tissue studies

Diagnosis was confirmed either by cytology (from CSF or lesions), histopathology (from a surgical biopsy or necropsy), or by a combination of them. The tissues were sampled for cytology by fine needle aspirate (FNA) and/or imprint. Data regarding the method of diagnosis and results were recorded. Diagnosis of lymphoma was based on the observation of a population of morphologically abnormal lymphoid cells.

The CSF analysis was considered abnormal if the results were above the reference values of nucleated cell count (NCC) ≤5 cells/μL, total protein (TP) concentration for cisternal puncture ≤25 mg/dL and lumbar puncture ≤45 mg/dL, respectively, and/or abnormal cell morphology or distribution. An increase in TP with normal NCC was defined as albuminocytological dissociation. On cytology, a diagnosis of lymphoma was considered if a population of lymphoid cells with atypical morphology was observed, which can include increased cell size (>2.5 times the upper limit of normal), irregular shape, and pointed borders of the cytoplasm, and nuclear atypia with any degree of sharp nuclear notches (13). When the diagnosis was based on cytology, only the samples with atypical large cells were considered in order to avoid possible diagnostic errors which could be induced by the morphological overlapping between small neoplastic lymphocytes and reactive lymphocytes (8, 14).

When possible, further classification was done based on the Revised European–American Lymphoma (REAL) classification of lymphoid neoplasms adopted by the World Health Organization (WHO) (15, 16). According to cell morphology large, intermediate or small cell tumor types were established and used to classify the tumor types in grades according to the National Cancer Institute working formulation (NCI WF) (16). The most recently published data on animal nervous system tumors was also considered (17).

On histology, the data recorded included: morphology of the cells, mitotic count/40X (low 0–1, moderate 2–4, and high ≥5), presence of degenerative changes (necrosis and/or hemorrhage), and tissue infiltration. Results of immunohistochemistry (IHQ) were also recorded and, when performed, tumors were classified as B-cell or T-cell lymphoma according to immunoreactivity of the cells in front of CD20 and CD3 markers, respectively, following published protocols (18).

Lymphomas were categorized into low, intermediate, or high grades (16). Small cell lymphoma cells had nuclei 1–1.5 times the diameter of the red blood cells whereas in large cell lymphoma nuclei cells were 2–3 times the diameter of the red blood cells. The small-cell lymphomas, including the small lymphocytic lymphoma, small-cleaved cell lymphoma, small lymphocytic lymphoma with plasmacytoid differentiation, and the small lymphocytic lymphoma with intermediate differentiation, form the low-grade lymphoid neoplasms. The large-cell and large cleaved-cell lymphomas form the intermediate-grade grouping. The high-grade lymphomas include lymphoblastic and small non-cleaved-cell lymphomas.

When complete necropsy examination was available the presence of lesions involving other organs (multicentric lymphoma) or not (primary lymphoma) was recorded.

### Statistical analysis

The characteristics from the MRI studies were retrieved to obtain explanatory variables. Data were arranged into contingency tables and the frequency distribution of the variables was assessed by a chi-square goodness of fit test using a R software. Results were considered significant if *p*-value was <0.05. In order to correlate the MRI features to the histological findings, the presence of necrosis was chosen as the dependent variable as this parameter was previously used for this purpose (12), and it can be related to the tumor grading, with higher grade lymphomas generally showing a higher incidence of necrosis (16). The explanatory variables were converted to binary and a Stata probit model was run to study the correlation. Results were considered significant if *p*-value was <0.05.

## Results

### Study population and clinical data

A total of 27 cats were included in the study. Results are summarized in Table 1.

**Table 1:** Signalment and clinical data of 27 cats with spinal lymphoma.

**Breed**	**Sex**	**Age, median** **(range)**	**FeLV test** **(*n* = 21)**	**Duration of signs** **(*n* = 21)**	**Initial signs**	**Clinical signs at presentation**
DSH 24/27 Maine Coon 2/27 Turkish Van 1/27	Male 13/27 Female 14/27	5 years (5 months−15 years)	Positive 9/21 Negative 12/21	≤7 days 12/21 8 days-1 month 7/21 1–3 months 2/21	Lameness 1/27 Progressive paresis 26/27	Spinal pain 9/27 Neurological sings 27/27: – ambulatory paraparesis 10/27 – non-ambulatory paraparesis 7/27 – paraplegia 6/27 – tetraparesis 2/27 – thoracic limb monoparesis 1/27 – tetraplegia 1/27

Breeds included domestic shorthair (*n* = 24), Maine Coon (*n* = 2), and Turkish Van (*n* = 1). Age ranged between 5 months and 15 years with a mean age of 5.9 years and a median of 5 years. In this group of cats, 11/27 (40.7%) had ages comprised between 5 months and 2.5 years, and 12/27 (44.4%) comprised between 8 and 15 years, the remaining 4 cats were aged 4 (2 cases), 5 and 6 years. Fourteen cats were females and 13 cats were males. FeLV test results were recorded for 21 cats, results were positive for 9/21 cats (42.9%) and negative for 12/21 (57.1%).

The time-lapse between the onset of the clinical signs and the MRI study was retrieved for 21 cats. For 12/21 (57.1%) the duration was ≤7 days, for 7/21 (33.3%) ranged from 1 week to 1 month, and for 2/21 (9.5%) of cases the duration of the signs was 3 months. The mean was 17.6 days (range 1–90 days).

According to the anamnesis, the owner's initial presenting complaint was signs of thoracic or pelvic limb lameness for 1/27 (3.7%) cats and progressive paresis for 26/27 (96.3%) cases (paraparesis 23/27, 85.2%, tetraparesis 2/27, 7.4% and monoparesis 1/27, 3.7%). The clinical signs on evaluation at the referral centers included spinal pain in 9/27 (33.3%) cats, ambulatory paraparesis in 10/27 (37%), non-ambulatory paraparesis in 7/27 (25.9%), paraplegia in 6/27 (22.2%), tetraparesis in 2/27 (7.4%), thoracic limb monoparesis in 1/27 (3.7%), and tetraplegia in 1/27 (3.7%) of cases.

Previous use of corticosteroids (prednisone or prednisolone) was recorded for 12 cats and of NSAIDs for 8 before diagnosis. In all the cases a temporary partial improvement was observed except in one cat from the NSAIDs group.

### MRI features

MRI studies were performed using high-field 1.5-Tesla magnets in 11 cats, a medium-field 0.5-Tesla magnet in 2 cats, and low-field 0.2-Tesla magnets in 14 cats.

The localization and characteristics of the lesions are summarized in Tables 2, 3.

**Table 2:** MRI distribution and pattern of spinal lymphoma in 27 cats on presentation.

	**Number (%)**	**Localization**	**Extense (+1 vert)**	**Spinal cord compression**	**T2W**	**T1W**	**Contrast enhancement**	**Enhancement pattern**
Epidural focal	12/27 (44.4)	Thoracic 5/12 Lumbar 6/12 Cervical 1/12	8/12	Moderate 7/12 Severe 5/12	Hyper 8/12 Iso 3/12 Iso-hyper 1/12	Iso 7/12 Hyper 4/12 Iso-hyper 1/12	Intense 6/12 Moderate 2/12 Mild 3/12 None 1/12	Homog 7/11 Heterog 4/11
Epidural multifocal (3 lesions)	1/27 (3.7)	Lumbar 3/3	1/3	Mild 2/3 Moderate 1/3	Hyper 3/3	Iso 3/3	Mild 3/3	Homog 3/3
Epidural and intramedullary multifocal (2 lesions)	1/27 (3.7)	Epidural: connus-Cc1 intramedullary: L2-conus	2/2	Mild 1/1	Hyper 2/2	Iso 2/2	Intense 2/2	Homog 1/2 (epidural) Heterog 1/2 (intramed.)
Epidural/intradural/ in tramedullary	1/27 (3.7)	Lumbar 1/1	0/1	Mild 1/1	Hyper 1/1	Iso 1/2	Intense 1/1	Heterog 1/1
Intramedullary multifocal (2 lesions)	1/27 (3.7)	Thoracic 2/2	2/2	-	Hyper 2/2	Iso 2/2	Mild 1/2 Intense 1/2	homog 2/2
Nerve roots intracanal	5/27 (18.5)	Cervical 2/5 Lumbar 2/5 lumbar-S3 1/5	4/5	Moderate 3/5 Severe 2/5	Iso-hyper 1/5 Hyper 4/5	Iso 3/5 Iso-hyper 2/5	Intense 4/5 Moderate 1/5	Homog 3/5 Heterog 2/5
Intramedullary and nerve roots (2 lesions)	1/27 (3.7)	Cervical 2/2	0/2	Mild 1/1	Hyper 2/2	Iso-hyper 2/2	Mild 2/2	Heterog 2/2
Paravertebral mass and epidural focal	4/27 (14.8)	Thoracic 4/4	3/4	Moderate 1/4 Severe 3/4	Iso 1/4 Iso-hyper 1/4 Hyper 2/4	Iso 4/4	Mild 1/4 Moderate 2/4 Intense 1/4	Homog 2/4 Heterog 2/4
Epidural and paravertebral mass multifocal (2 lesions)	1/27 (3.7)	Epidural: L5-S1 Paravertebral T12-L3	2/2	Moderate 1/2 Severe 1/2	Hyper 2/2	Iso 2/2	Mild 2/2	Heterog 2/2
Total	Focal 22/27 (81.5) Multifocal 5/27 (18.5)	Cervical 5/33 (15.15) Thoracic 12/33 (36.36) Lumbar-lumbosacral 16/33 (48.48)	22/33	Mild 5/29 Moderate 13/29 Severe 11/29	Hyper 26/33 Iso 4/33 Iso-hyper 3/33	Iso 24/33 Iso-Hyper 5/33 Hyper 4/33	None 1/33 Mild 12/33 Moderate 5/33 Intense 15/33	Homog 18/32 Heterog 14/32
Extraneural involvement								
– Bone	7/27 (26)							
– Soft tissue	11/27 (40.7)							

Hyper, hyperintense; Iso, isointense; Homog, homogeneous; Heterog, heterogeneous.

**Table 3:** MRI distribution and imaging pattern of spinal lymphoma on follow-up MRI performed in 4 cats.

	**Localization**	**Treatment**	**Time between MRI**	**Extense (+1 vert)**	**Spinal cord compression**	**T2W**	**T1W**	**Contrast enhancement**	**Extraneural findings**
Case 1 1st MRI	Epidural half L2 to L3-L4 lateralized to right	Surgery	18 days	Yes	Moderate	Iso	Iso	Intense homog.	Bilateral sublumbar muscle enhancement
2nd MRI	Epidural half L2 to half L4 lateralized to left			Yes	Severe	Iso-hyper	Iso	Intense homog.	
Case 2 1st MRI	Epidural L4-L5 lateralized to the left	Surgery	13 months	No	Severe	Iso-hyper	Hyper	Intense heterog.	None
2nd MRI	Paravertebral lumbosacral right, L7 nerve root			No	Mild	Iso-hyper	Hyper	Intense heterog.	Right iliac crest, L6 and L7 vertebrae
Case 3 1st MRI	Epidural T8-T10 left	Surgery and chemotherapy	3 months	Yes	Moderate	Iso	Iso	None	None
2nd MRI	Epidural 2 lesions: T8 to T9 right and L3 right			Yes No	Mild Mild	Iso Iso	Iso Iso	Mild homog. Mild homog	none
Case 4 1st MRI	Epidural connus-Cc1 and intramedullary L2-conus	Chemotherapy	3 months		Mild	Hyper	Iso	Intense epidural homog. Intramedullary heterog.	L7 vertebral
2nd MRI	Malacic lesion L5				None			None	None

Hyper, hyperintense; Iso, isointense; Homog, homogeneous; Heterog, heterogeneous.

On the first presentation, an epidural solitary lesion was present in 12/27 (44.4%) cats. The lesion was localized in the cervical vertebral column in 1/12 cases, the thoracic vertebral column in 5/12 cases, and the lumbar vertebral column in 6/12. In 3 of these 12 cases, a second MRI (2 after surgery and 1 after surgery and chemotherapy) was performed due to recurrence of signs, and new lesions with differences in location and extension were found. Results are summarized in Table 3. In 1/27 (3.7%) cases multifocal epidural lesions were present (at T13-L1, L4-L5, and L5-L6 vertebrae).

**Figure 1:**
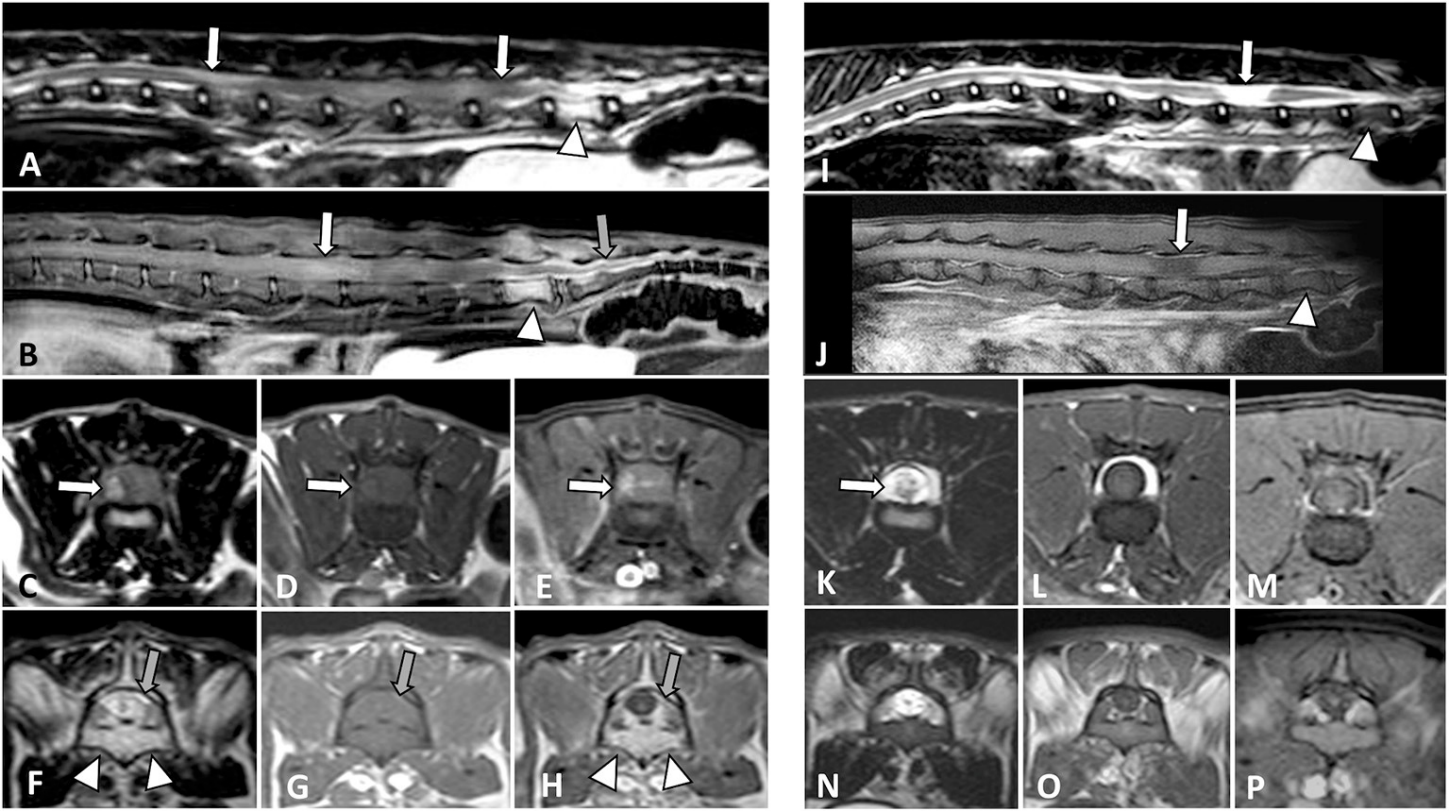
MRI studies of a 1-year-old domestic short-haired paraplegic cat. A multifocal large cell lymphoma with an intramedullary lumbosacral lesion and a sacrococcygeal epidural lesion was diagnosed. On T2W sagittal plane **(A)** the spinal cord is swollen with an increased heterogeneous signal from L2 to L6 vertebrae (arrows). On T1W post-contrast SPIR sagittal plane **(B)** there is a diffuse intramedullary contrast enhancement (white arrow) and an intense epidural enhancement from L7 to the first coccygeal vertebrae (gray arrow). On transverse planes at the level of L4-L5 vertebrae, the spinal cord appears swollen due to an intramedullary lesion with a right component which is hyperintense on T2W **(C)**, isointense on T1W **(D)**, and shows heterogeneous enhancement on post-contrast SPIR **(E)** images. On transverse planes at the level of the L7 vertebra, there is an epidural infiltrative lesion hyperintense on T2W and isointense on T1W images (gray arrows in **F,G**, respectively) with intense post-contrast enhancement (gray arrow in **H**). An increased signal of the L7 bone marrow with cortical sparing is observed (arrowheads in **A,B,F,H**). After chemotherapy and clinical improvement, an MRI was performed at 3 months **(I–P)**. On the sagittal planes (**I**, T2W; **J**, T1W post-contrast SPIR) the L7 bone marrow signal was normalized (arrowheads) and a malacic lesion, presumptively residual, was present along the L5 vertebra (arrows). On transverse planes at the level of L4-L5 vertebrae (**K**, T2W; **L**, T1W and **M**, T1W post-contrast SPIR), the previous intramedullary lesion was not observed, and signs of malacia were seen (arrow in **K**). The corresponding L7 vertebra transverse planes **(N–P)** did not show epidural changes.

Two separate lesions, one epidural extending from the conus medullaris to the first coccygeal vertebrae and another intramedullary lesion extending from the L2 vertebra to the conus medullaris were found in 1/27 (3.7%) cases (Figures 1A–H). The cat was treated with chemotherapy (COP protocol) with improvement, in a control MRI that was done 3 months later a residual intramedullary malacic lesion on the L5 vertebra was the only relevant finding (Figures 1I–P) (case 4 in Table 3). At 9 months, an MRI of the head was done due to recent diffuse intracranial signs, showing a diffuse and intense meningeal enhancement compatible with lymphomatous meningitis. Further studies were not allowed by the owners.

A single lesion with intramedullary/intradural/epidural involvement (located at L5-L6 vertebrae) was present in 1/27 (3.7%) cases and two separate intramedullary lesions (located at T2-T4 vertebrae and T9-T11 vertebrae) were present in 1/27 (3.7%) cat.

**Figure 2:**
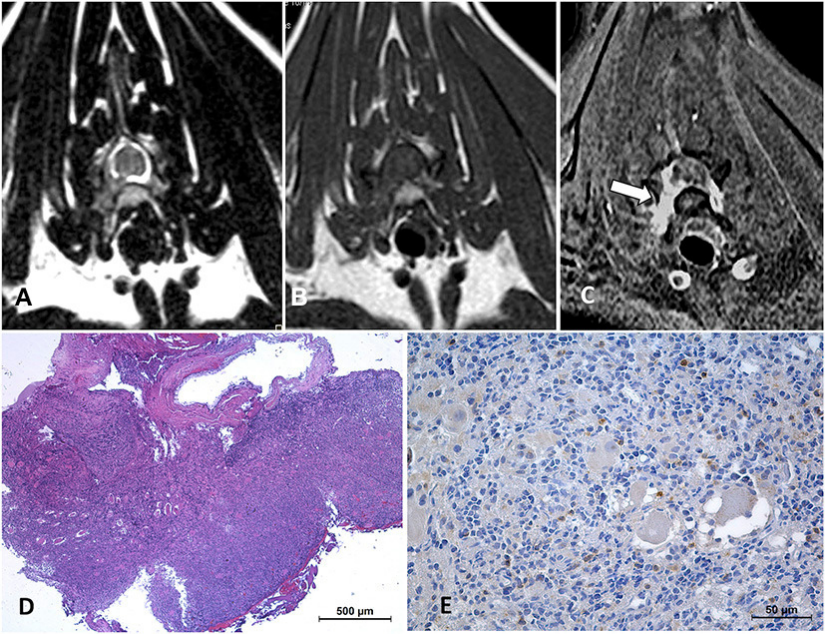
Cervical neurolymphomatosis in a 2.5-year-old domestic short-haired cat which presented with a 3-month-course of right thoracic limb lameness progressing to monoparesis. In the MRI transverse planes at the level of C6-C7 intervertebral foramina, there is a diffuse enlargement of the right C7 spinal nerve and root with mild spinal cord contralateral displacement (arrows on **A–C**). The lesion is iso-hyperintense on T2W images **(A)**, isointense on T1W **(B)**, and enhances with an intense and heterogeneous pattern on post-contrast SPIR images **(C)**. On biopsy tissue **(D)** neoplastic lymphoid cells are infiltrating adjacent tissues, HE. CD 20-immune positive cells predominate in the neoplastic lymphoid cell population **(E)**. The final diagnosis was neural and ganglionar small non-cleaved B-cell lymphoma.

**Figure 3:**
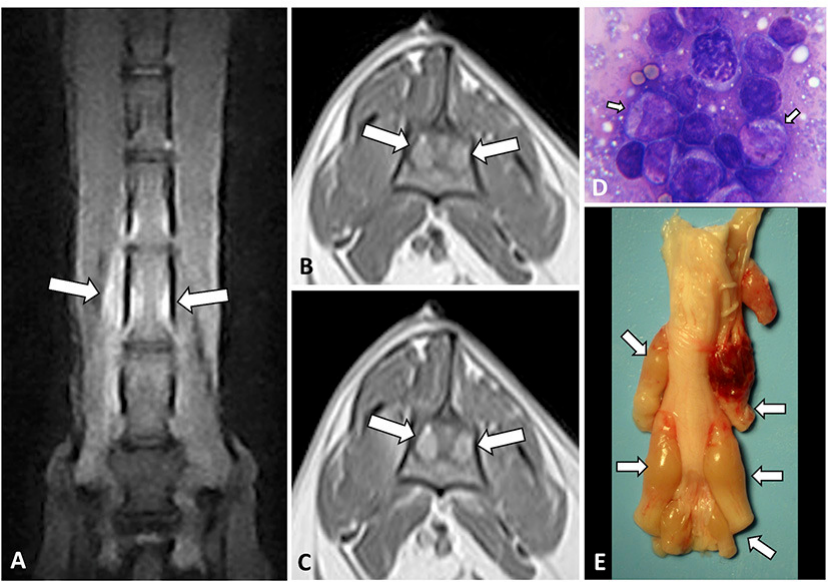
Dorsal STIR **(A)** and transverse T1W pre- **(B)** and T1W post-contrast **(C)** images of the lumbar vertebral column of an 11-year-old Turkish Van cat with progressive non-ambulatory paraparesis. There is bilateral diffuse nerve root enlargement (arrows), which shows hyperintense on STIR **(A)**, mildly hyperintense on T1W **(B)** and heterogeneously enhancing **(C)**. A surgical biopsy smear **(D)** showed an abnormal population of pleomorphic lymphoid cells, with immature large lymphocytes with more abundant cytoplasm and pale chromatin (arrows), Romanowsky, 100x. Intraoperative euthanasia was elected, on the macroscopic sample **(E)** the nerve roots from L5 to sacral spinal cord segments were enlarged and swollen (arrows).

Lesions involving nerve roots and causing spinal cord compression were present in 5/27 (18.5%) cases, localized in the right C5 to C8 nerve roots, the right C6-C7 nerve roots (Figure 2), bilateral L5-L7 nerve roots 1/5, right L6 and left L7, and bilateral L5-S3 (Figure 3), respectively. An intramedullary lesion located at C4-C5 and a separate lesion involving the right C6-C7-C8 nerve roots was observed in 1/27 (3.7%) cases.

**Figure 4:**
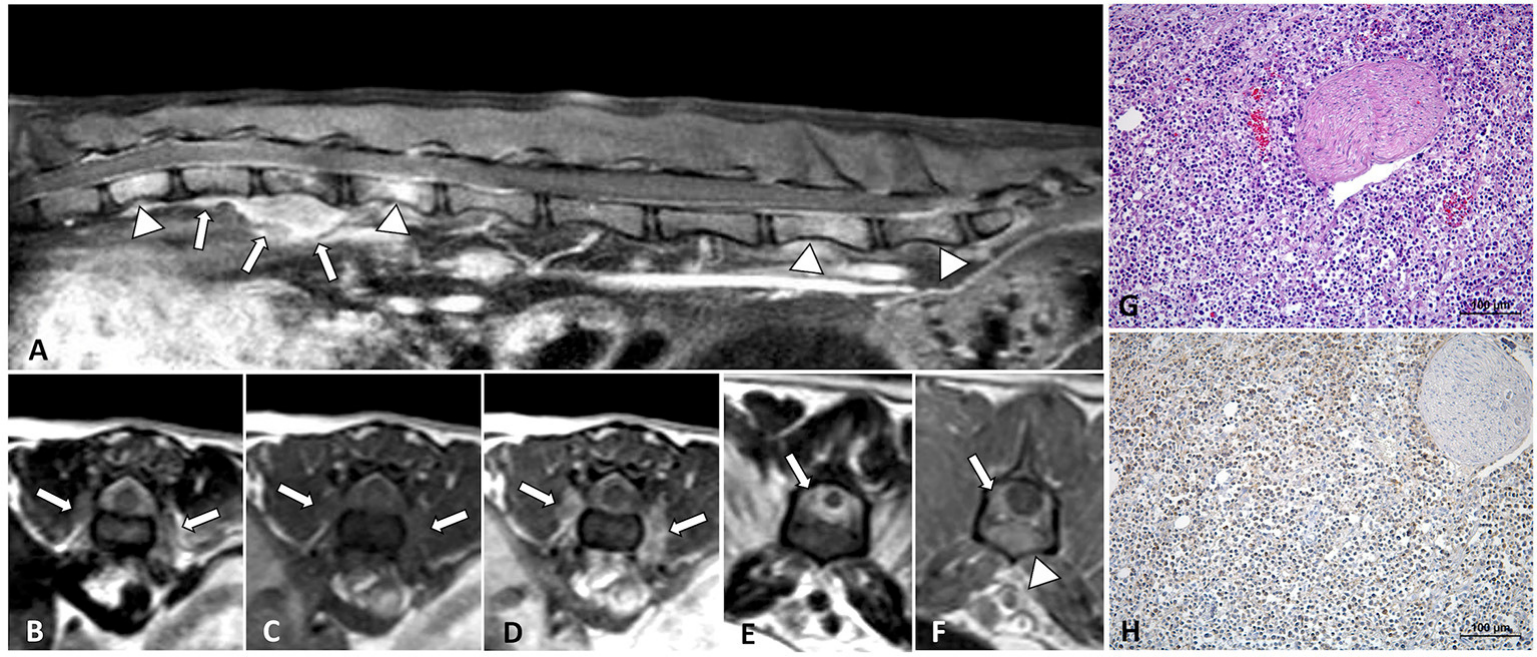
MRI of an 8-year-old domestic short-haired paraplegic cat diagnosed with multicentric small-intermediate T-cell lymphoma. Sagittal plane T1W post-contrast SPIR of the caudal thoracic and lumbar vertebral column **(A)** shows a paravertebral mass ventral to the L1 vertebra and extending cranially to T12 and T13 vertebrae (arrows) and irregular hyperintensity on the bone marrow of several vertebral bodies (arrowheads). Transverse planes at the level of T13-L1 intervertebral space **(B–D)** show an extension of the paravertebral mass through the intervertebral foramina (arrows) and to the epidural space circumferential to the spinal cord. The lesion is hyperintense on T2W **(B)**, isointense on T1W **(C)**, and enhances with a mild heterogeneous pattern on post-contrast T1W **(D)** images. Transverse planes T2W **(E)** and post-contrast T1W **(F)** at the level of the L6 vertebra, show an epidural diffuse infiltrate (arrows) with similar signal features. Hyperintensity of the vertebral body with cortical sparing is also depicted (arrowhead in **F**). On histopathology image, HE **(G)** neoplastic lymphoid cells are surrounding a nerve root, on IHC **(H)** T-lymphocytes, CD3-immune positive cells, predominate in the tumor.

A paravertebral mass with vertebral canal invasion along nerve roots was found in 4/27 (14.8%) cases, localized at T2-T4 vertebrae, T5-T7, T8-T9, and T6-T12 vertebrae, respectively. In 1/27 (3.7%) case a paravertebral mass with vertebral canal invasion along nerve roots in T12-L3 vertebrae and a separate epidural lesion in L5-S1 vertebrae were depicted (Figure 4).

In 22/27 (81.5%) cases a focal lesion was found, and 5/27 (18.5%) cases had multifocal lesions. Considering the 3 cases with recurrence in the follow-up studies lesions were focal in 24/30 (80%) and multifocal in 6/30 (20%).

A total number of 33 lesions were observed in the 27 patients on presentation, and 4 new lesions were observed in the follow-up MRI studies thus making a total of 37 lesions. Lesions were present at the level of the lumbar and lumbosacral (19/37, 51.35%), thoracic (13/37, 35.13%), and cervical (5/37, 13.51%) vertebrae. Extensive lesions (larger than 1 vertebral body) were present in 24/37 (64.86%) cases. Lesions were circumferential to the spinal cord in 13/37 (48.15%), lateralized to the right in 11/37 (29.73%), lateralized to the left in 9/37 (24.33%), and non-lateralized in 4/37 (10.8%) cases.

All the lesions were ill-defined. In relation to the spinal cord, lesions were intramedullary in 4/37 (10.81%) cases, extramedullary in 32/37 (86.48%) cases, and both intra- and extramedullary in 1/37 (2.7%) case. Spinal cord compression associated with the extramedullary lesions was mild in 8/33 (24.24%), moderate in 13/33 (39.39%), and severe in 12/33 (36.36%) cases.

**Figure 5:**
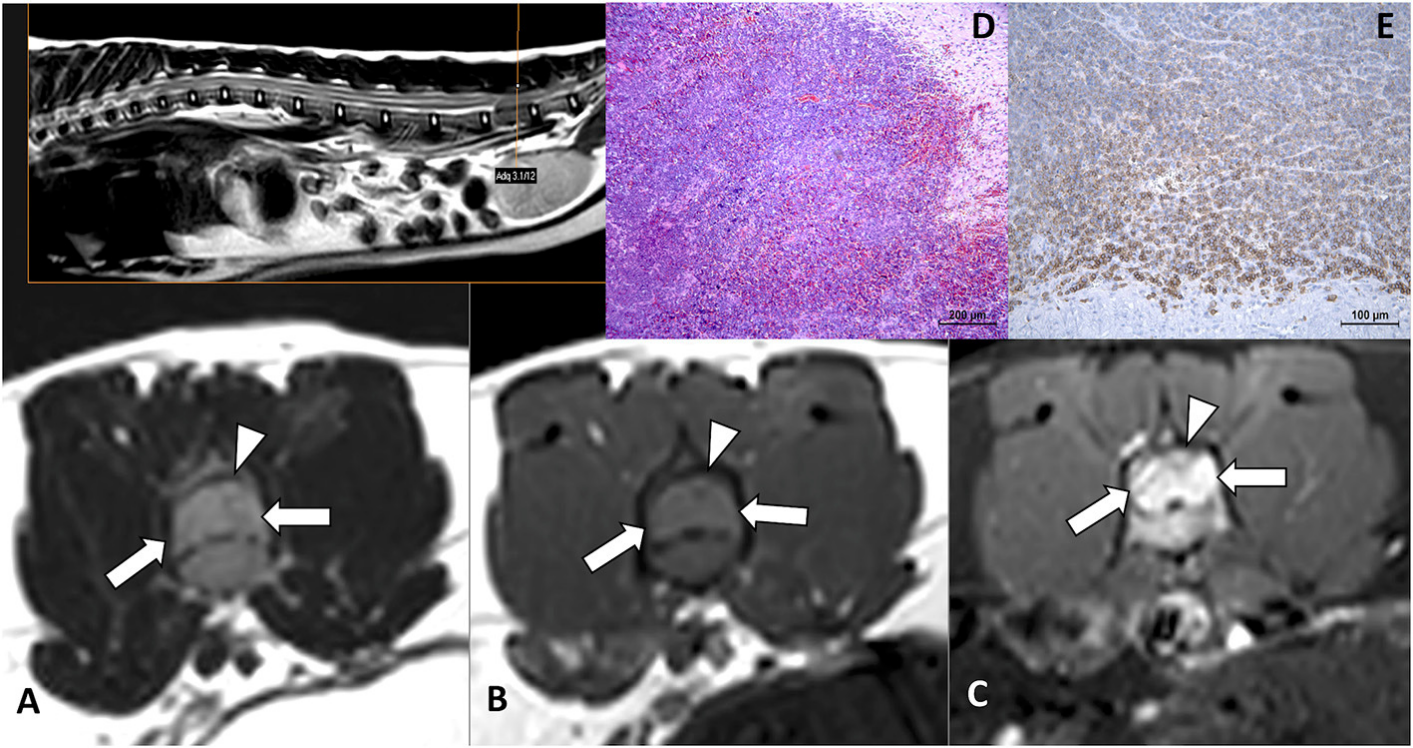
Intermediate-large B-cell lymphoma in an 8-month-old domestic shorthair cat with a history of progressive paraparesis and caudal lumbar spinal pain for 10 days. On MRI transverse planes at the level of L6 **(A–C)**, there is an epidural lesion isointense on T2W (arrows in **A**) and T1W (arrows in **B**) images. On T1W post-contrast SPIR **(C)** there is an intense enhancement of the lesion (arrows) as well as diffuse vertebral bone marrow enhancement. The spinal cord appears severely compressed with dorsal displacement (arrowheads in **A–C**). On HE stain of tissue biopsy **(D)**, there are neoplastic lymphoid cells, mixed with erythrocytes, infiltrating nervous tissue. B lymphocytes, CD20-immune positive cells, predominate in the tumor in IHC **(E)**.

**Figure 6:**
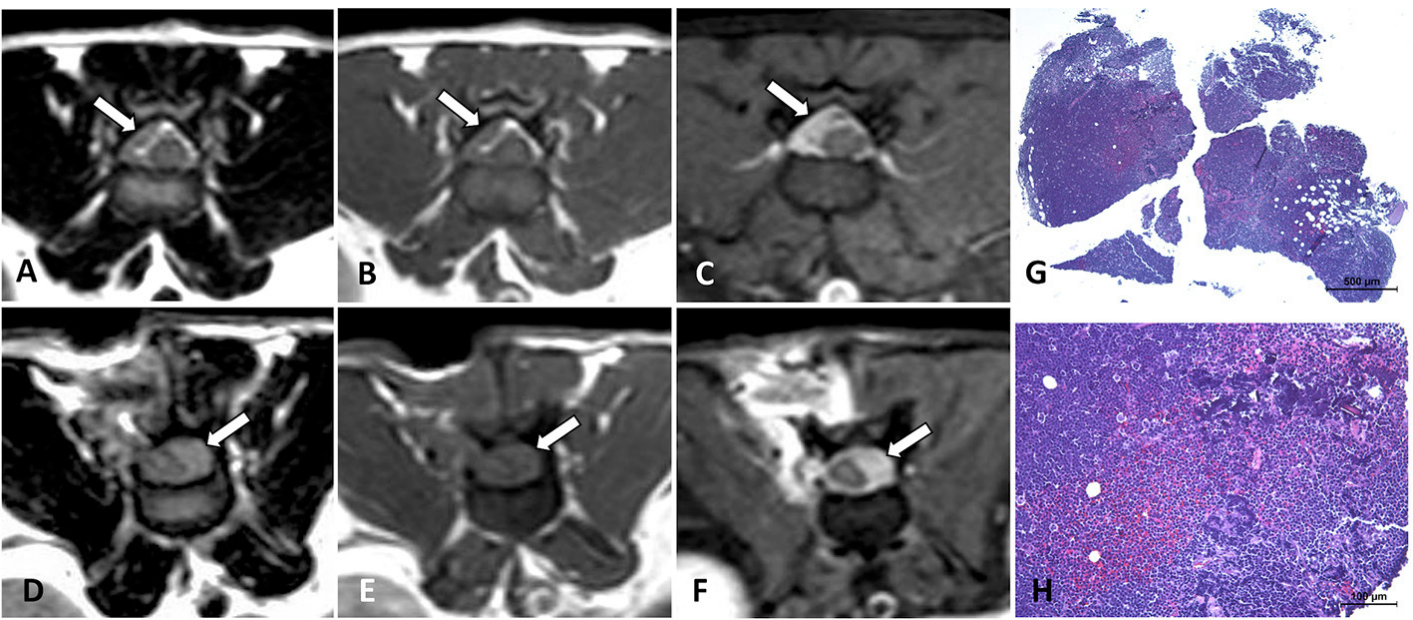
MRI of a 4-year-old domestic short-haired cat with progressive ambulatory paraparesis for 7 days, diagnosed with large B-cell lymphoma. Transverse MRI of a cat at the level of the cranial portion of the L3 vertebral body **(A–F)**. There is an epidural dorsolateral right mass (arrows on **A–C**) isointense on T2W **(A)** and T1W **(B)** images, which shows intense enhancement on post-contrast T1W SPIR image **(C)** and induces mild spinal cord contralateral displacement. The images **(D–F)** correspond to the same plane and sequences as **(A–C)**, respectively, 18 days later after a right hemilaminectomy for decompression and tissue sampling was performed. A left epidural lesion was found (arrows on **D–F**) inducing a greater spinal cord compression. Tissue biopsy **(G,H)** HE, neoplastic lymphoid cells including basophilic (dark blue) necrotic foci and mixed with infiltrating erythrocytes.

On relaxometry, lesions were hyperintense (26/37, 70.27%) isointense (6/37, 16.21%), or iso-hyperintense (5/37, 13.51%) relative to the spinal cord on T2W sequences. Isointensity on T2W images was seen in cases with solitary epidural lesions in 5/6 (Figures 5A, 6A) and 1/6 cases with paravertebral mass with epidural extension. The 5 iso-hyperintense lesions corresponded to 2/5 epidural, 1/5 nerve roots, and 2/5 paravertebral mass. A case with an epidural isointense lesion had an increased signal with iso-hyperintensity on a follow-up MRI (case 1 in Table 3; Figure 6). On T1W sequences lesions were isointense (27/37, 72.97%), iso-hyperintense (5/37 13.51%) or hyperintense (5/37, 13.51%). Iso-hyperintensity on T1W sequences corresponded to lesions in nerve roots 3/5, intramedullary 1/5, and epidural 1/5, and hyperintensity was observed in 4/5 epidural lesions and 1/5 paravertebral mass. In the last group, the paravertebral mass corresponded to the follow-up of a case formerly epidural which maintained the same relaxometry.

STIR sequences were obtained in 14/27 cases. All of them showed a hyperintense and heterogeneous signal of the lesions (Figure 3A) and corresponded with hyperintensity on T2W images in all cases. Additional diffuse hyperintensity of bone was observed in 2 cases on presentation (affecting the L1 vertebral body in one, and L5 and L6 vertebral bodies in other) and in 1 case in the follow-up (involving the right iliac crest, and the L6 and L7 vertebral bodies case 2 in Table 3).

On T1W sequences after contrast administration, fat saturation (SPIR) was applied to 11/27 studies. Enhancement was not observed in 1/37 (2.7%) lesions, for the rest enhancement was intense (17/37, 45.94%), mild (14/37, 37.84%), or moderate (5/37, 13.51%). In the only case with 2 intramedullary lesions enhancement was mild in one of the lesions and intense in the other, in the remaining cases if lesions were multifocal enhancement was similarly observed in all. The non-enhancing lesion corresponded to a case with an epidural focal lesion that had previously received corticosteroids. In this case (case 3 in Table 3) when a second MRI was performed 3 months after surgery and chemotherapy the 2 new epidural lesions observed showed mild contrast enhancement.

Regarding the pattern of contrast enhancement it was homogeneous in 21/36 (58.33%) lesions and heterogeneous in 15/36 (41.66%) lesions. In the only case that an epidural lesion and a separate intramedullary lesion, the first enhanced homogeneously whilst enhancement was heterogeneous in the intramedullary lesion (Figures 1B,E,H).

Additional soft tissue involvement, apart from the paraspinal masses, was found in 11/27 (40.7%) cases. These included 12 lesions, encompassing the paraspinal soft tissues contiguous to the lesions in 8/12 (66.6%) and distant soft tissue abnormalities in 4/12 (33.3%) with renal and intestinal enhancement (1/12, 8.3%), hepatomegaly (1/12, 8.3%) and iliac adenomegaly (2/12, 16.6%), respectively. When present, the relaxometry of the paraspinal soft tissue lesions was similar to the vertebral canal lesions.

Local bone involvement was present in 7/27 (25.93%) cases, affecting multiple vertebrae and the sacrum in 2/27 (7.41%) cases, and only one vertebra in 5/27 (18.52%) cases. In addition, in one case, involvement of the L6 and L7 vertebrae and the right iliac wing was only present in the follow-up MRI (case 2 in Table 3). When the bone was involved the MRI pattern was characterized by diffuse signal changes and contrast enhancement in the bone marrow with cortical sparing (Figures 1A,B,F–H, 5A–C) in all cases except one case (corresponding to an epidural focal lesion) in which a small focus of cortical signal loss was observed with overall vertebral shape preservation.

### CSF analysis

CSF data were collected from 12/27 cases, results are summarized in Table 4. In one case the sample was obtained by lumbar puncture and for the rest by cerebellomedullary cistern puncture.

**Table 4:** Results of CSF analysis and cytopathological studies in 27 cats with spinal lymphoma.

	**CSF** **(12/27)**	**FNA** **(9/27)**	**Biopsy** **(12/27)**	**Necropsy** **(6/27)**	**Cell** **morphology**	**Mitotic count**	**Necrosis**	**IHQ**	**Pathological diagnosis**
Epidural focal (12/27)	-	1	-	-	Large	-	-		Large cell
	-	1	-	-	Large	-	-		Large cell
	-	1	-	-	Large	-	-		Large cell
	-	1	-	-	Large	-	-		Large cell
	-	-	1	-	Large, pleo morphic	Moderate	Yes	B-cell	Large B-cell
	-	-	-	1	Interm, pleo morphic	Low	-	-	Intermediate cell
	-	1	-	-	Interm/large, pleomorph	-	-	-	Interm/large cell
	ACD	-	1	-	Interm/large, monomorp	Moderate	-	-	Interm/large lymp
	Normal	-	1	-	Interm/large, pleomorph	Low	-	B-cell	Interm/large B-cell
	-	-	1	-	Small	High	Yes	T-cell	Small non-cleaved T-cell
	Normal	-	-	1	Interm-large, monomorp	Low	-	-	Lymphoblastic
	-	-	-	1	Small, mon omorphic	High	-	-	Small non-cleaved cell
Total	3	5	4	3					
Epidural multifocal (1/27)	–	1	1	-	Large	Low	-	-	Epitheliotropic T-cell
Epidural and intramedullary multifocal (1/27)	Neoplastic cells	-	-	-	Large	-	-	-	Large cell
Epidural/intradural/ intramedullary (1/27)	Neoplastic cells	-	-	-	Large, pleomorphic				Large granullar cell
Intramedullary multifocal (1/27)	↑NCC, ↑TP	-	-	1	Pleomorph, occasional plasmacytoid differentation	High	Yes		Leptomeningeal lymphoplasmacytic lymphomatosis
Spinal nerve roots/intracanal (5/27)	Neoplastic	-	-	-	Large	-	-	-	Large cell
	Neoplastic	-	-	-	Large	-	-	-	Large cell
	-	-	1	-	Large	-	-	-	Large cell
	-	-	1	-	Interm-large, pleomorph	-	-	-	Interm/large
	↑NCC	-	1	-	Small	Low	-	B-cell	Small non-cleaved B cell
Intramedullary and nerve roots multifocal (1/27)	↑NCC, ↑TP	-	1	–	Medium-large, pleomorph	High	-	T-cell	Interm/large
Paravertebral mass and epidural focal (4/27)	↑NCC, ↑TP	1	1	1	Large	High	Yes	T-Cell	Large granular T-cell, multicentric
	-	1	1	-	Large, monomorphic	High	-	-	Large cell
	-	1	-	-	Large, pleomorphic	Low	-	-	Large cell
	ACD	-	1	-	Small-interm, pleomorphic	Low	-	T-cell	Small/interm T-cell, multicentric
Epidural and paravertebral mass multifocal (1/27)	-	-	-	1	Small/interm, pleomorphic	Moderate	Yes	T-cell	Multicentric lymphoma
Total	Normal 2/12 Abnormal 6/12 Diagnostic 4/12				Small: 2 Small-medium: 2 Medium: 1 Medium-large: 6 Large: 14				

CSF, cerebrospinal fluid; NCC, nucleated cell count; TP, total protein; ACD, albuminocytologycal dissociation; FNA, Fine needle aspirate.

Cell count and/or cytology were abnormal in 8/12 cases. NCC count was elevated in 7/12 cats (range 6–547 cells/microl, mean 224.5 cells/microl). Differential cytology was abnormal in 6/12 cats. Cell count and cytology were normal in 4/12 cases.

A cell population different from 100% lymphocytes was found in 4 cases. Neutrophils were present in 2 cases (with 37 and 96%, respectively), and activated monocytes were present in 2 cases (7 and 2%, respectively). Morphologically abnormal lymphocytes were observed in 5 cases, with activated lymphocytes present in one case and a population of large neoplastic lymphocytes in 4 cases, one of them further categorized as large granular cell lymphoma.

TP was determined in 10 cases and was elevated in 8/10. Specific measurements ranged from 30 to 300 mg/dl (mean value 103.4 mg/dl) in the samples retrieved from cervical cisternal puncture and determination was 100 mg/dl in the sample obtained from a lumbar puncture. Albuminocytological dissociation was present in 3 cases.

Corticosteroids were used previously to CSF sampling in 6/12 cats, in 1/6 results were normal, in 2/5 NCC was elevated in the lower limit (6 and 9 cells, respectively) and 3/5 corresponded to albuminocytological dissociation.

### Cytopathological and histopathological studies

Pathological studies were performed in samples obtained from the lesions by FNA (9/27), tissue biopsy (12/27), necropsy (6/27), or a combination of them. Details about cell morphology, mitotic count, presence of necrosis, and IHQ are shown in Table 4.

In the 9 cases in which FNA was performed the sample was taken from epidural masses in 6/9 cases and from perivertebral affected tissue in 3/9 cases. Additional FNA of a cutaneous nodule was performed in 1 case. In 9/9 results were compatible with lymphoma with a high population of morphologically atypical lymphoid cells. In the case sampled from an epidural lesion and a cutaneous nodule, results were similar from both samples. A further description was available in one case with a paravertebral mass that was characterized as a presumptive large granular cell lymphoma and later confirmed by necropsy.

A surgical tissue biopsy was performed in 12/27 cases. From vertebral canal masses in 11/12 [epidural localization 5/12 (Figures 5, 6), paravertebral with vertebral canal invasion 2/12, or nerve roots 4/12] including surrounding tissues (bone, epidural fat), and a paravertebral lesion in 1/12. An additional biopsy of a cutaneous nodule was obtained in 1 case. For the cases with epidural lesions infiltration to bone was reported in 3 of them.

Cytology from an intrasurgical lesion smear was performed in 3/12 cases (Figure 3D) with results compatible with lymphoma in all, in one case further categorization as intermediate-large cell was achieved by histopathological studies.

A lymphocytic proliferative population of cells was present in all samples. IHQ was performed in 8/27 cases, in 5/9 there was immunopositivity for T-cells and in 3/9 for B-cells.

Ten cats were euthanized either after MRI diagnosis or in the following days due to rapid clinical worsening, corresponding to epidural focal (3/10), epidural multifocal (1/10), epidural/intradural/intramedullary (1/10), nerve roots (2/10), paravertebral mass (2/10) and epidural and paravertebral mass (1/10) localizations. One cat with multifocal intramedullary lesions died in the following days after diagnosis.

Anatomopathological studies were performed in 6/27 cases. On 2/6 multicentric involvement of other organs (liver, spleen, lungs, and/or intestines) was observed. The studies included 3/6 cases with epidural localization based on MRI, 1/6 intramedullary, 1/6 paravertebral mass with vertebral canal invasion, and 1/6 multifocal case with epidural and paravertebral masses with vertebral canal invasion (Figure 4).

In general, an infiltrative pattern was described in all the tissue samples, with the presence of necrosis and/or hemorrhage reported in 5 cases. Cell pleomorphism was reported in 10 cases. Mitotic count was recorded in 16 cases and described as low (7/16), moderate (3/16), or high (6/16). According to histopathology tumors were classified as low grade (1/27), intermediate grade (22/27), and high grade (4/27). Data are summarized in Tables 4, 5.

**Table 5:** Frequency of distribution in 27 cases of feline spinal lymphomas classified according to the National Cancer Institute working formulation (NCI WF).

**Grade**	**Tumor type**	**Number (%)**
Low	Lymphocytic plasmacytoid	1
	Total	1/27 (3.7)
Intermediate	Mixed cell	8
	Large cell	14
	Total	22/27 (81.5)
High	Small non-cleaved cell	3
	Lymphoblastic	1
	Total	4/27 (14.8)

### Diagnosis

Final diagnoses in this group of 27 cats were achieved by CSF (4/27, 14.8%), FNA (6/27, 22.2%), FNA and histopathology on surgical biopsy (2/27, 7.4%), cytology from smears on surgical biopsy (2/27, 7.4%), cytology from smear and histopathology on surgical biopsy (1/27, 3.7%), histopathology on surgical biopsy (6/27, 22.2%), necropsy (5/27, 18.5%), or CSF, FNA, surgical biopsy and necropsy (1/27, 3.7%).

### Treatment and outcome

Results from treatment and survival time are summarized in Table 6. In 8/27 cases surgery for decompression and tissue sampling for biopsy was performed and was followed by chemotherapy in 4/8. Chemotherapy alone was the treatment for 2/27 cases. Euthanasia was performed on the day of the MRI in 4/27 cases, and in <1 week in 6/27 due to worsening of the signs despite medical treatment, 1/27 died in the following days. Data from survival time since MRI longer than 1 week were available for 9/27 cats and ranged from 1 week to 13 months (mean 4.5 months), but 5/9 cats were lost to follow-up at that recorded time, therefore the last known survival time was considered for the study. For the rest of the 7/27 cats survival time is unknown as they were lost to follow-up after diagnosis.

**Table 6:** Treatment and survival time in 27 cats with spinal lymphoma.

	**Number (%)**	**Surgery**	**Chemo-therapy**	**Medical management**	**Euthanasia on the day of MRI**	**Euthanasia on the following days of MRI**	**Survival time (known)**	**Final pathological diagnosis**	**Grading (NCI WF)**
Epidural focal	12/27	-	-	1/2	-	?	?	Large cell	Intermediate
	(44.4)	-	-	1/2	-	?	?	Large cell	Intermediate
		-	-	1/2	-	?	?	Large cell	Intermediate
		-	-	1/2	-	-	1 week, LTF	Large cell	Intermediate
		1/12	-	1/2	-	-	2 weeks	Large B-cell	Intermediate
		-	-	-	1/2	-	-	Intermediate cell	Intermediate
		-	1/12	-	-	-	4 months, LTF	Interm/large cell, pleom	Intermediate
		1/12	1/12	-	-	-	3 months	Interm/large, monom	Intermediate
		1/12	1/12	-	-	-	1 month, LTF	Interm/large B-cell	Intermediate
		1/12	-	1/12	-	-	13 months	Small non-cleaved T-cell	High
		-	-	1/12	-	1/12	-	Lymphoblastic	High
		-	-	1/12	-	1/12	-	Small non-cleaved cell	High
Total		4/12	3/12	8/12	1/12	2/12			
Epidural multifocal (3 lesions)	1/27 (3.7)	-	-	1/1	-	1/1	-	Epitheliotropic T-cell (metastatic?)	Intermediate
Epidural and intramedullary multifocal (2 lesions)	1/27 (3.7)	-	1/1	-	-	-	12 months	Large cell	Intermediate
Epidural/intradural/intra-medullary	1/27 (3.7)	-	-	-	1/1	-	-	Large granullar cell	Intermediate
Intramedullary multifocal (2 lesions)	1/27 (3.7)	-	-	1/1	-	1/1 (death)	-	Leptomeningeal lymphoplasmacytic lymphomatosis	Low
Nerve roots intracanal	5/27 (18.5)	-	-	-	1/5	-	-	Large cell	Intermediate
		-	-	1/5	-	-	?	Large cell	Intermediate
		-	-	1/5	-	-	?	Large cell	Intermediate
		-	-	1/5	-	1/5	-	Interm/large cell	Intermediate
		1/5	1/5	-	-	-	1 month, LTF	Small non-cleaved B cell	High
Intramedullary and nerve roots (2 lesions)	1/27 (3.7)	1/1	-	-	-	-	?	Interm/large	Intermediate
Paravertebral mass and epidural focal	4/27 (14.8)	-	-	1/4	-	1/4	-	Large granular T-cell	Intermediate
		1/4	1/4	-	-	-	6 months, LTF	Large monom	Intermediate
		-	-	1/4	-	1/4	-	Large pleom	Intermediate
		1/4	-	1/4	-	-	?	Small/interm T-cell	Intermediate
Epidural and paravertebral mass multifocal (2 lesions)	1/27 (3.7)	-	-	-	1/1	-	-	Multicentric T-cell	Intermediate
Total		8/27	6/12	16/27	4/27	7/27			

NCI WF, National Cancer Institute working formulation; LTF, lost to follow up.

For the 2/27 cases treated with chemotherapy alone, survival time was 4 months (epidural intermediate/large cell lymphoma) and 12 months (epidural and intramedullary large cell lymphoma), respectively. For the 4 cases managed with surgery without chemotherapy survival time was recorded for 2/4 and was 2 weeks (epidural large B-cell) and 13 months (epidural small non-cleaved T-cell, at that time a new lesion with paravertebral location was diagnosed, case 2 on Table 3), respectively. For the 4 cases treated with surgery combined with chemotherapy time of survival was at least 1 month for 2/4 cats (epidural intermediate/large B-cell and small B-cell nerve roots neurolymphomatosis), 3 months for 1/4 (epidural intermediate/large cell, at that time MRI showed new epidural lesions, case 3 on Table 3) and at least 6 months for 1/4 (paravertebral large cell).

Data regarding survival time was available for 14 of the cases in which tissue was obtained either by biopsy or necropsy. In this group for the 5/14 cases that showed necrosis in the pathological studies, 4/5 (80%) died or were euthanized ≤1 month after the MRI. For the rest of the 9/14 cases that did not show necrosis 4/9 (44.44%) died or were euthanized ≤1 month after the MRI. Results are shown in Table 7.

**Table 7:** Survival time at 1 month in 14 cats with or without necrosis observed in tumoral tissue samples.

	**Survival time ≤1 month**	**Survival time > 1 month**	**Number (%)**
Necrosis	4/5 (80)	1/5 (20)	5/14 (35.7)
No necrosis	4/9 (44.44)	5/9 (55.55)	9/14 (64.3)
Number (%)	8/14 (57.14)	6/14 (42.86)	

### Statistical analysis

When data from all the MRI studies were retrieved explanatory variables were obtained regarding lesion localization (cervical, thoracic, lumbar/lumbosacral), lateralization (circumferential, right, left, no), extension (≤ or >1 vertebral body), compression degree (mild, moderate, severe), T2W signal (iso, iso-hyper, hyper), T1W signal (iso, iso-hyper, hyper), contrast enhancement (none, mild, moderate, intense), contrast enhancement pattern (homogeneous, heterogeneous), vertebral-bone involvement (present/absent) and extraneural findings (present/absent). In the chi-square goodness of fit test significant (*p*-value <0.05) values were obtained for a higher prevalence of lumbar/lumbosacral (*p*-value = 0.01171) and circumferential (*p*-value = 0.04906) localization, T2W hyperintensity (*p*-value = 4.364e-06), T1W isointensity (*p*-value = 8.998e-07), and bone involvement (*p*-value = 0.001177).

When the explanatory variables data from the MRI features were converted to binary and compared to the presence of necrosis on histopathology as a dependent variable, the only significant results were found for T2W imaging. A T2W hyperintensity was significantly correlated with the non-presence of necrosis (coefficient −2.084253, *p*-value = 0.017) when compared to T2W iso-hyperintensity. For T2W isointensity the estimator was not significant for a 5% confidence level therefore there do not seem to be differences when compared to iso-hyperintensity.

## Discussion

### Primary and secondary incidence

It has been reported that 90% of feline lymphomas affecting the CNS have a spinal cord localization (4), with an overall prevalence of primary lymphoma considered to be low (19), similar to the human counterpart (11). Spinal cord lymphoma is most commonly secondary, encountered as a part of a multicentric process, frequently with renal or bone marrow involvement (20). In one study multicentric involvement was present in 84.6% of cats with spinal cord lymphoma (2), and another study showed that although all cats were evaluated for neurologic deficits, 85% of the necropsied cases had a multicentric disease (21). Primary lymphoma, originated and restricted to the spinal cord, has been also described in cats with intramedullary and epidural localizations reported (6, 19, 22). In humans, almost 100% of primary spinal cord lymphomas affect intramedullary structures and the majority of metastatic lymphomas are extramedullary (6). It is worth remarking that in previous literature epidural lymphomas have been often grouped with CNS lymphomas, however, considering that the epidural space does not belong to the CNS, the term CNS lymphoma applied for general cases or cases affecting the epidural space should be avoided.

In the present study distant lesions on abdominal organs were depicted in 4/27 of the cases, compatible with multicentric lymphoma. Alterations were present on MRI in 2 cases, on necropsy in 1 case, and in the other case, MRI findings of multicentric pattern were confirmed by necropsy (Figure 4). Due to the low number of complete necropsies (5/27), the actual incidence of multicentric lymphoma cannot be inferred but may be greater, especially taking into account the previous studies. It is also possible, as it has been pointed out in another study (6) that some cases could be secondary although no other sites of lymphoma were detected in the diagnostic work-up, which would explain the predominantly extramedullary or combined intra- and extramedullary distribution of the lesions in our group. On the other hand, it should be considered that feline lymphomas, when localized in the spinal cord, can be primary.

### Age and sex

A bimodal tendency was observed in the present study with 40.7% of cats ≤2.5 years and 44.4% of them aged between 8 and 15 years. Spinal cord lymphoma was traditionally considered to be a juvenile neoplasm in cats (4, 21) but has been reported as well as one of the most common spinal cord diseases in cats > 2 years of age, with median and mean ages of 4.5 and 6.3 years, respectively (1). A bimodal age has been reported before in feline lymphoma, with a group of cats younger than 3 years and a group of cats older than 8 years (23). A recent report showed similar results with a wide age distribution ranging from 1 to 20 years, with a median age of 14 years (7). In light of the strong bimodal distribution, neither the mean nor median value depicts a real age predisposition.

No sex predisposition was found in this study, as male and female cats were similarly affected. A study of incidence and risk factors for all types of feline lymphoma found that male cats had an increased risk compared to females similar to other studies, but others, as well as the present study, have failed to report an association between lymphoma and sex (24).

### Relation to FeLV

In this study, FeLV test results were negative for 57.1% and positive for 42.9%. This prevalence is in between the previously referenced values, which shows variations depending on the studies. Cats have been reported to be positive for FeLV in 17.6% of cases in a study of feline intracranial lymphoma in 2003 (25) and in 56.5% of cases in a study of spinal cord lymphoma in 2008 (2). Since 1990 a decrease in the prevalence of FeLV infection in cats with lymphoma has been described, probably related to vaccination and population control programs (20, 26), and nowadays the majority of cats with lymphoma are unaffected by FeLV so the clinical importance of this factor is comparatively low (9). In the study by Meichner et al. in (27), only 13% of cats with lymphoma were FeLV antigen-positive. However, Mello et al. reported in 2019 a rate of cats with NS lymphoma infected with FeLV of 87.5% similar to proportions presented prior to the 1990s, which may reflect differences in prevalence in different regions of the world.

The bimodal age has been related to FeLV in feline lymphoma, with cats younger than 3 years widely associated with FeLV infection and cats older than 8 years generally free from infection (23). The same tendency seems to be present in this study, as for the group of younger cats most of them (63.6%) were FeLV positive and for the group of older cats the majority (83.3%) were negative.

### Clinical signs

In this group of cats, the presentation was generally acute to sub-acute with rapid progression. For most of the cats (90.4%) clinical signs were present from 1 day to 1 month with the majority of them (57.1%) having a duration of ≤7 days. For the rest, the duration of signs did not exceed 3 months.

The most common reported initial clinical sign, affecting 96.3% of all the cats, was progressive paresis (85.2% paraparesis, 7.4% tetraparesis, 3.7% monoparesis). Besides, lameness was the initial complaint in 3.7% of cases.

The clinical signs observed when cats presented at the referral centers included signs of spinal pain (33.3%) and neurological deficits referred to a spinal cord localization most commonly affecting the pelvic limbs (85.1%). Other signs included tetraparesis (7.4%), thoracic limb monoparesis (3.7%), and tetraplegia (3.7%).

Similar presenting signs have been previously reported, with a duration of <60 days (2). The rapid progressive neurological deterioration, in some cases after an initial insidious course, was in concordance with other reports (4).

### MRI distribution and pattern

In our group of 27 cats with spinal lymphoma, an epidural solitary lesion was the most common localization at presentation (44.4%). Other localization patterns varied and mostly included vertebral canal nerve roots (18.5%) or a paravertebral mass with vertebral canal invasion (14.8%), with other localizations less commonly seen. When the 27 cases plus the 3 follow-ups that had lesions were considered together, lesions were focal in the majority of the cases (80%), for the rest, a multifocal localization with more than one lesion affecting the nervous system was observed. Multifocal lesions affecting different spinal cord compartments were found in 6.66% with localizations epidural and intramedullary and intramedullary and nerve roots. All the vertebral canal masses had indistinct (ill-defined) margins. Extensive vertebral canal lesions spreading more than 1 vertebral body were present in 64.86% but this variable was not statistically significant (*p* > 0.05).

Similarly, previous reports have described the MRI characteristics of spinal cord lymphoma in cats showing similar features to those seen in dogs (7) and to the intracranial counterpart (6), as lesions commonly indistinctively marginated from the surrounding tissue, mostly with a focal (80.6%) and extensive (64.5%) pattern (6, 8). Others have also reported spinal lymphoma as focal, from 76% (21) to 95.6%, presented as a single lesion that may expand over more than one vertebral body, or multifocal, as two or more separate lesions (4). In a report of 8 cats with spinal cord lymphoma (23) the pattern was also often focal (75% of cases). In a similar way, it has been reported a tendency of spinal cord lymphoma to form extensive lesions (21) with most tumors extending over multiple vertebral bodies, and 19% with more than 1 level of spinal cord involvement. Other studies had reported that in one-half of the cases spinal lymphoma extends to other spinal cord segments (2, 5).

The selection of cases of the present study implied vertebral canal invasion, and in relation to the spinal cord lesions were most frequently extramedullary (86.48%), followed by intramedullary (10.81%), and less frequently both intra- and extramedullary (2.7%). The degree of spinal cord compression of extramedullary lesions most commonly ranged from moderate (39.39%) to severe (36.36%). Previously, lymphoma of the spinal cord has been reported as extradural, intradural–extramedullary, intramedullary, or with a combination of these compartments (20).

A previous study (2) revealed extradural and intradural components in 29 of 33 (87.9%) cats with lymphosarcoma, with 34.5% having an exclusive extradural location and 10.3% an exclusive intradural location. When extradural, lymphoma has been described typically as an epidural mass resulting in spinal cord compression, which can extend into and through the adjacent vertebrae as well as into the underlying muscle (7). The lesions may have a variable mass effect from mild to moderate (2, 6, 7). Similarly to our study, it has been reported predominantly situated extradural to the spinal cord (20, 23), moreover, a study found that when the lesion was centered on epidural soft tissues, it was more likely lymphoma (7) and rarely occurs as an intramedullary tumor (2, 8). On the other hand, in a study of 92 cats with clinical signs of spinal cord disease (3) 3/7 of cats diagnosed with spinal lymphoma had intramedullary lesions. Intradural-extramedullary lymphoma occurs as a non-delineated mass replacing the subdural and subarachnoid spaces, and commonly infiltrating the spinal cord parenchyma or nerve roots (1, 20), but the pattern of intradural infiltration without an epidural mass was not found in our study. In a recent study (8) lesions frequently affected multiple spinal compartments similar to previous publications (1, 2) conversely, although intradural-extramedullary involvement with epidural lesions could be observed on MRI, both intra- and extramedullary involvement were present only in one lesion on this study. In humans, an epidural location for lymphoma is observed in 0.1–6.5% of all lymphomas (11).

Regarding localization in relation to the vertebral column, lesions were significantly most frequent in the lumbar and lumbosacral areas (51.35%, *p*-value < 0.05). Other localizations included the thoracic (35.13%) and cervical (13.51%) segments. In previous studies, spinal cord lymphoma exhibited a predilection for lumbar (1, 8, 23) and thoracic regions (4, 8) or both (2, 7, 20). It has been observed that as lumbosacral spinal cord segments are generally affected by lymphoma, this is frequently associated with more spinal cord region involvement (1, 20) but this tendency was not found in this report. In humans, the most common region of involvement is the thoracic vertebral column, followed by the lumbar and cervical vertebral column (11).

Nerve root involvement was present in 22.2% of cases, with cervical (50%), lumbar (33.33%), and lumbosacral (16.66%) localizations. In one of the cervical cases, an intramedullary lesion was also present, thus the case was considered multifocal. It has been previously described that lymphoma may involve the spinal cord from vertebral canal lesions centered on the nerve roots or from a vertebral canal extension along peripheral nerves (8, 20, 28), but lymphoma of the peripheral nerves, named neurolymphomatosis, has been considered sporadic in cats, and less frequently with vertebral canal invasion and spinal cord involvement either by compression or/and infiltration of the subarachnoid space and spinal (4, 8, 21, 26). Neurolymphomatosis of the lumbosacral plexus, although similar to our study less commonly reported than in the brachial plexus, was the most common lesion pattern in a group of cats (8). MRI findings (8, 29) of lumbosacral lymphoma have been described with symmetrical or asymmetrical thickening and enhancement of the nerve roots or/and nerves, meningeal enhancement of the conus medullaris, with or without the presence of an extradural, intradural, or intramedullary mass, or some combination of these. Conversely, in the study from Durand et al. that included 8 cats with spinal cord and spinal nerves lesions, >50% of the cases showed lesions involving both, which was more common than the present and previous reports.

A paravertebral mass with vertebral canal invasion was observed in 4/27 (14.8%) cases, with 3/4 located in the thoracic segments and 1/4 thoracolumbar. In the latter, a multifocal presentation with a separate epidural lumbosacral lesion was observed (Figure 5). Spinal lymphoma with paravertebral soft tissue involvement in the form of paraspinal masses has been previously reported (20) but not frequently described on MRI. In the study of Durand et al., the only case described corresponded to a cat with multifocal hypaxial mass and epidural lesions. In the study of Auger et al., in a group of 20 dogs and 5 cats, round cell tumors were centered on paraspinal soft tissues in 8% of cases, which is less than in our study.

Interestingly, in the cases in which MRI was repeated new lesions with different locations and extensions were found. Therefore, it may be concluded that recurrence of spinal lymphoma with the appearance of different localizations is possible.

Regarding the patterns of signal intensity, on T2W sequences the lesions showed mainly hyperintense (67.57%, *p*-value < 0.05) isointense (18.91%), or iso-hyperintense (13.51%). On T1W sequences the most prevalent signal was isointense (72.97%, *p*-value < 0.05), the remaining lesions showed iso-hyperintense (13.51%) or hyperintense (13.51%). On STIR sequences lesions were hyperintense, corresponding to the T2W sequences. In a follow-up study (Figure 6) the new lesion had an increased signal on T2W images compared to the previous lesion.

In previous studies, a general preponderance of hyperintensity on T2W sequences and isointensity on T1W sequences has been reported as well. In a study (7) the percentage of T2W hyperintense extradural lesions was even higher and on T1W images lesions were predominantly isointense (62.5%) or either hyperintense in a higher percentage (37.5%), but this study included feline and canine spinal lymphomas. Similarly, a study with 2 cases (6) described the lesions as T2-hyperintense and T1-hypointense in both intramedullary and epidural lesions, and in another study (8) the lesions were also described as hyperintense in T2W and isointense on T1W. The other less common mixed patterns of relaxometry found in this study, iso-hyper on T2 or T1 have not been reported before.

Enhancement of the lesions after contrast administration was variable, and appeared as intense (45.94%), moderate (13.51%), or mild (37.84%). In the only case with 2 intramedullary lesions enhancement was mild in one of the lesions and intense in the other, but in the remaining cases if lesions were multifocal enhancement was similarly observed in all. Contrast enhancement was not observed only in one case with an epidural focal lesion. Regarding the pattern of contrast enhancement, it was either homogeneous (58.33%) or heterogeneous (41.66%). Previous reports showed that in general, contrast enhancement of the lesion is present and has been described as homogeneous (6, 7, 20), but it can be faint to none (8). The absence of enhancement has been also described in humans, as primary CNS lymphoma can present as longitudinally extensive transverse myelopathy and may be non-contrast enhancing on gadolinium-enhanced MRI (30). Our study shows similar results, as lesions most commonly enhanced homogeneously, but not significatively different from a heterogenous pattern. A previous study reported that in cases with multifocal lesions, these lesions showed overall similar MRI characteristics (8), this observation is similar to our study, although one case showed 2 separate epidural and intramedullary lesions with homogeneous and heterogeneous enhancement, respectively. A characteristic contrast enhancement for spinal lymphomas was not found in this study due to the variable degree and pattern.

It has been reported that corticosteroids may reduce or eliminate abnormal contrast enhancement which may complicate the interpretation of enhanced MRI (12, 13). Marked reduction in enhancement or clinical improvement following corticosteroid therapy, although seen in other conditions, has been suggested to be highly suspicious for CNS lymphoma (13). In a study of radiographic findings of primary CNS lymphoma in humans nearly all the lesions enhanced, except after corticosteroid administration (10). In our study, 12 cats had received corticosteroids before imaging and all except one of this group showed contrast enhancement, therefore although corticosteroids may have influenced the degree of intensity, contrast enhancement of the lesions was generally observed. In fact, the non-enhancing lesion corresponded to a case that had previously received corticosteroids. In this case (case 3 in Table 3) when a second MRI was performed 3 months after surgery and chemotherapy the 2 new epidural lesions observed showed mild homogeneous contrast enhancement. Our study is the first to report follow-up MRI studies, and therefore the first to report a change in the degree of enhancement.

Extraneural soft tissue involvement, apart from the paraspinal masses, was found in 40.7% of cases with a total of 12 lesions, affecting mainly the paraspinal soft tissues contiguous to the lesions (66.6%) but also with distant soft tissue abnormalities (33.3%) with renal and intestinal enhancement (8.3%), hepatomegaly (8.3%) and iliac adenomegaly (16.6%), respectively. When present, the relaxometry of the paraspinal soft tissue lesions was similar to the vertebral canal lesions. In one case with an epidural focal lesion, extraneural soft tissue involvement appeared only in the follow-up MRI (case 2 in Table 3). Different from our study, extra-neural tissue invasion was previously reported to be less common (16.7%) in cats with spinal lesions (8). On the other hand, abdominal lymph nodes were the most frequently affected in secondary lymphomas in another study (23), and the presence of abdominal lesions has been reported to be more likely in patients with round cell neoplasia (7).

Local bone involvement was present in 26% of cases at presentation, affecting multiple vertebrae and the sacrum (7.41%) or only one vertebra (18.52%). In addition, in one case, involvement of the L6 and L7 vertebrae and the ilion was only present in the follow-up MRI. Bone involvement was centered in the bone marrow and characterized by diffuse signal changes and contrast enhancement, better depicted on STIR and post-contrast fat-saturated sequences. Cortical sparing was typically maintained, in only one case a small focus of cortical signal loss was observed but anyway with overall vertebral shape preservation. In cats diagnosed with lymphoma in previous studies (7, 20, 23), some cats had polyostotic bone lesions with cortical sparing on MRI, with multifocal sites of extradural spinal cord compression, whereas others had lesions centered on the epidural soft tissues with no evidence of bone involvement. A previous study (8) has shown a similar characteristic pattern in lymphoma, with absent or mild cortical lysis with preserved vertebral shape. This pattern is similar in dogs (7) and humans (31). In the study from Auger et al. bone lesions in cats and dogs showed different patterns of signal intensity on T1W and T2W and diffuse STIR hyperintensity. Failure of bone marrow suppression on STIR sequences has been reported in dogs with spinal lymphoma (32) and has been attributed to the replacement of normal bone marrow fat by malignant neoplastic cells, inflammation, and/or necrotic tissue. In agreement with previous studies performed in dogs, a STIR sequence, either in the sagittal or dorsal plane, should be part of the imaging protocol of the vertebral column, especially in low-field magnets and if round cell neoplasia is a consideration (32, 33). On high field magnets, T1W contrast-enhanced fat-suppressed images demonstrate similar patterns to STIR (34).

In conclusion, as well as in humans (11, 30) a presumptive antemortem diagnosis of spinal lymphoma can be established based on MRI data.

### CSF and tissue studies

In our group of cats, the antemortem diagnosis was reached in 81.5% of cases, which is above than reported before, and was achieved either by CSF (14.8%), FNA (22.2%), surgical biopsy (37%), or by a combination of them. In a study of feline lymphoma including intracranial, spinal, and spinal nerves lesions (8), antemortem diagnosis was achieved in 32.3% of cases, and similarly to our study was based on CSF cytology (4/10), histopathology on biopsy (3/10), tissue cytology (2/10), or a combination of cytology and histopathology (1/10). In the same study lymph node FNA was performed in 4 cats, being diagnostic for lymphoma only in 1 cat which had diagnostic CSF cytology, therefore lymph node FNA did not add relevant information for diagnosis. Also, a lower percentage of antemortem diagnosis was reported in another study of 85 cases of tumors affecting the spinal cord of cats with an antemortem diagnosis of lymphoma reached in 21.2% of cases (2). In another study, the diagnosis of CNS lymphoma was based on the identification of lymphoblasts in CSF in 3 dogs and 2 cats, and necropsy in 5 dogs and 2 cats (6).

### CSF

In our study, all the CSF parameters were normal only in two of the cases in which it was analyzed. Differential cytology was abnormal in 50% of cats and in 33% of cases neoplastic large cells were observed therefore allowing a diagnosis. In one case small lymphocytes were observed, but it was not possible to give a definitive diagnosis based on cytology morphology. As the differentiation between small neoplastic and reactive lymphocytes may be difficult in feline lymphoma (14) additional test as flow cytometry (13) or Polymerase chain reaction for antigen receptor rearrangement (PARR) (8), may assist in the diagnosis but those were not performed in the present study.

Total NCC count was elevated in 58% of cases with neoplastic lymphocytes observed in 3 of them. It has been reported that CSF was diagnostic for lymphoma in the cases with higher NCC counts which is in agreement with the previously reported difficulty in diagnosis in CSF with low cells number (13). In a study of inflammatory CSF in 62 cats (35), CSF analysis alone was useful in the diagnosis of cats with lymphoma. In this study, all cats had mononuclear inflammation, and in 5 cats large atypical lymphoid cells were observed in the CSF. Similarly to our study, total NCC was variable (6–1,144 cells/μl).

TP was determined in 10 cases and was elevated in 80% with a wide range of 30–300 mg/dl. A wide variation has been reported as well in cats with lymphoma with TP levels varying from normal to elevated (31–1,000 mg/dl) (35). Albuminocytological dissociation was present in 30% and corresponded to 2 cats with epidural lesions and 1 cat with a paravertebral mass.

CSF examination remains a good diagnostic option in cases of lymphoma with intradural invasion (20), with a diagnosis supported by the identification of large neoplastic lymphocytes (6, 14). It is worthwhile to point out that in the veterinary literature, it has been common to refer to the largest malignant lymphocytes as lymphoblasts, whereas in human pathology, the term lymphoblast is used to describe an immature cell of small size, larger than a mature lymphocyte and smaller than a large lymphocyte, and this nomenclature should also be used (16).

Close to our results, in a previous study neoplastic lymphocytes diagnostic of feline spinal lymphoma were identified on CSF analysis in 35% of cats (4). Others (8) have reported that CSF was abnormal in 80% and diagnostic in 20% in a group of cats with NS lymphoma, pleocytosis associated with increased TP was most commonly observed (65%), and similar to our study albuminocytological dissociation was present in 35%. In another study, the evaluation of CSF in 11 cats with lymphosarcoma revealed large neoplastic lymphocytes in 1 cat, albuminocytological dissociation in 2 cats, and an increase in protein content and neutrophilic pleocytosis in 2 cats (2). Additionally, in cases of neurolymphomatosis CSF examination might be useful in demonstrating a lymphocytic pleocytosis (20, 28), in fact, in our group of 5 cats with lesions centered on the nerve roots CSF was retrieved in 3 cases and was diagnostic in 2 of them.

In humans, CSF examination is included in the recommendation of workup in patients with primary epidural lymphomas (11). A review of the systematic approach to the diagnosis of suspected CNS lymphoma (13) concluded that at least one of the routine CSF indices is abnormal in more than 80% of cases at the time of diagnosis. CSF cytology can provide definitive diagnostic information in CNS lymphoma, and with the aid of immunohistochemical studies, it is possible to identify atypical lymphoid cells as monoclonal (neoplastic). As well as in animals, there is some overlap in the morphologic features of neoplastic and inflammatory lymphocytes, and this can make the interpretation of cytology difficult. The same study indicates that the sensitivity of CSF cytology varies widely (2–32%).

It has to be considered that the previous use of corticosteroids may have influenced the CSF analysis results, especially regarding the NCC. Also, the sensitivity of CSF cytology is reduced after exposure to corticosteroids (13). In the present study, in the 6 cats previously treated with corticosteroids in which CSF was sampled the result was normal in 1/6, the number of cells was elevated but low in 3/6 (range 6–9 cells/microL) and albuminocytological dissociation was found in 2/6. In these cases, the NCC could have been lowered by the corticosteroid effect. The small amounts of the samples generally obtained from cats may have also influenced the ability for diagnosis. In humans, sensitivity improves when a larger volume (≥10.5 mL) is analyzed but this is not feasible in cats. Again in humans, when CSF results are inconclusive repeated CSF examinations are indicated for definitive diagnosis (13, 30, 36). Serial CSF samples should be considered in cases with compatible MRI findings if FNA of the lesion/s is not possible, and also as a less invasive procedure than surgical biopsy.

According to the present and previous (8) studies, the MRI features when combined with CSF analysis may provide an antemortem diagnosis in cats with spinal lymphoma.

### Cytopathological and histopathological studies

In the case of a non-diagnostic CSF, FNA or surgical biopsy of the lesion/s may be of greater diagnostic value than CSF alone (14). In the present study pathological studies were performed, either alone or combined, in samples obtained from the lesions by FNA, imprints, or tissue samples either by biopsy or necropsy. In the 4 cases in which tests were combined, the same diagnosis was supported and further categorization in tumor types was achieved in 2 of them. For the rest, a single test was able to provide a diagnosis of lymphoma.

In all the samples obtained by FNA, a high population of morphologically abnormal lymphoid cells compatible with lymphoma was observed. A further description was available only in one case that was characterized as large granular lymphocyte lymphoma (and later confirmed by necropsy), therefore, FNA of the lesions allowed the confirmation of lymphoma but generally, typification was not achieved. FNA can be obtained from the tumoral mass, more easily when a paravertebral soft tissue mass or enlarged nerves are identified by imaging, and/or from other masses of affected organs if detected (35, 37). Small cell lymphomas are rare but can be suspected by FNA (14) and when necessary differentiation from inflammatory lymphocytes can be supported by PARR. In humans, FNA is commonly used for NS lymphoma diagnosis. In a study addressing paraspinal masses in 59 cases, FNA yielded a definitive diagnosis in 66% of the cases and tissue biopsy yielded no additional information. The sensitivity and specificity of FNA of paraspinal masses were 88 and 75%, respectively (38).

In surgical tissue biopsies, a lymphocytic proliferative population of cells was present in all samples. When cytology from an intrasurgical lesion smear was performed it was compatible with lymphoma in 25% of cases, and further categorization was achieved in one case based on histopathological studies. Therefore, similarly to FNA samples, a diagnosis of lymphoma was established by lesion smears but a further classification was not obtained. Surgical tissue samples are good candidates for histological investigation (2) and it has been reported that a preliminary diagnosis can be facilitated by intraoperative smear cytology (20). Similarly in humans, the different tumor types are not discernable by neuroimaging, therefore histological evaluation of biopsied tissue is commonly necessary for the definitive diagnosis of neurotropic lymphoma. Macroscopically, extradural lymphoma typically appears as a focal soft, gray, non-encapsulated collection of neoplastic tissue in the epidural fat, that may invade the dura mater and spinal cord parenchyma and locally extend into the vertebral bodies or the underlying soft tissue (20). When intradural-extramedullary, lymphoma occurs as an irregular mass within the subdural and subarachnoid spaces and commonly infiltrating the spinal cord parenchyma or nerve roots (1, 20). Surgical nerve biopsies directed by imaging are also valuable in suspected cases of neurolymphomatosis (28). In humans, lymphoma affecting primarily peripheral nerves is very rare, and diagnosis is based on MRI and surgical biopsy (37).

In postmortem anatomopathological studies of the case with an MRI localization of intramedullary multifocal, an infiltrative leptomeningeal lymphoplasmacytic lymphomatosis was diagnosed, showing a more extensive infiltration than the imaging study, involving spinal roots and nerves. For the rest of the studies, MRI depicted the extension of the lesion infiltration in bone and soft tissues corresponding to the pathological findings. Leptomeningeal lymphomatosis is characterized by widespread leptomeningeal infiltration by neoplastic lymphocytes (39) and has been reported in cats (20). Neoplastic lymphocytes invade the leptomeningeal space by direct extension from preexisting primary or metastatic CNS tumors or infiltrate through arachnoid vessels created by the tumor. Lymphomatous meningitis may be difficult to detect grossly (20), and in this study was not detected by MRI. Necropsy allowed the confirmation of multicentric involvement in a case that was suspected by MRI.

IHQ studies were performed in order to improve the characterization of the tumors in 8 cases with a result of 62.5% T-cell lymphomas and 37.5% B-cell lymphomas. Immunocytochemistry can be used to detect specific antigens on a cell's surface which allows the differentiation of cell lines and of neoplasms subtypes, such as the B- and T-cell subtypes of lymphoma (14). According to Harris et al. immunophenotyping was helpful in some diagnoses of lymphoma, but not required for many others. In general, in the absence of immunophenotyping, those large cell lymphomas with basophilic cytoplasm tend to be of B-cell type, and those with water-clear cytoplasm tend to be of T-cell type (16). The morphological and histopathologic distribution of nervous system lymphoma in cats has been previously described (20, 23). The REAL classification of lymphoid neoplasms adopted by the WHO Harris, was adapted for animals and applied for the classification of feline lymphomas in an attempt to correlate histotypes and biological behavior (16, 23), this classification is based on both morphology and immunophenotype, and on cytogenetics for some subtypes. This system requires immunophenotyping and does not provide a low/high grading system (16) and moreover, and as IHQ was not available for all cats in the present study, the NCI WF system was used to classify lymphoma subtypes based on histological studies as low, intermediate and high-grade. Most of the tumors were categorized in the intermediate-grade (81.5%), followed by the high-grade (14.8%) and only one (3.7%) was considered a low-grade lymphoma.

The clinical significance of anatomical and cytomorphological evaluation in feline lymphomas has been indicated before in a study (40), which reported that cats with globule leukocyte lymphoma showed significantly shorter survival than those with high-grade and other low-grade lymphomas and cats with high-grade lymphomas showed significantly shorter survival than cats with other low-grade lymphomas. The incidence of different tumor types varies greatly between studies. In one study (21), lymphomas were high-grade lymphoblastic or immunoblastic types in all cats. In the study of Valli et al., about 30% of cat lymphomas were of intermediate grade and about half were of high grade. Only about 2% of cats had lymphoblastic lymphomas in the lower categories of mitoses, and those classified as high-grade lymphomas were more frequent than expected in the higher categories of mitoses. In a study on non-neurological feline lymphoma, there was no cytomorphological predominant subtype (40).

### Outcome

In our group of cats, 40.74% of them did not survive more than 1 week after diagnosis. It has to be considered that data from survival time since MRI longer than 1 week were only available for 9/27 cats and ranged from 1 week to 13 months, but as 5/9 cats were lost to follow up at that recorded time the actual survival time for this group is unknown but may be greater.

Survival time was recorded for 2 cases treated with chemotherapy alone and for 2 cases only surgically treated, showing a wide variation (from 2 weeks to 13 months). Therefore, due to the low and dispersed numbers, a correlation between treatment and survival was considered difficult to establish for those groups. For the 4 cases treated with surgery combined with chemotherapy time of survival ranged from 1 to 6 months (mean 2.75 months). A previous study (21) reported that a single cat treated with laminectomy and post-operative chemotherapy had a prolonged remission (15.5 months). In our group of cats that were similarly treated, the mean survival time was lower but it has to be taken into account that 2 cats were lost at 1 month and this may have lowered the actual survival time. In humans, when primary spinal epidural lymphoma is suspected surgery is indicated to ameliorate the spinal cord compression and to facilitate a specimen for histological diagnosis. Surgery may be then followed by chemotherapy/radiotherapy (11).

It has been reported that the prognosis for cats and dogs with brachial plexus lymphoma is unknown (41) but one study of peripheral nerve sheath tumors in dogs reported that it is worsened when there is spinal cord involvement (42). In our study, cats were selected only if the neurolymphomatosis invaded the spinal canal, therefore a worse prognosis compared to an extracanal localization was expected.

When the cases were grouped in grades according to the NCI WF (Table 6) the was no apparent correlation with the survival time. In general, larger survival times corresponded to intermediate grading, but there were exceptions (the only case in the low grade died in the following days of MRI and one case with signs of necrosis on histopathological studies had the largest survival time with surgical treatment alone). However, a correlation was found between survival time and the presence of necrosis in histopathological studies, with lower survival times when necrosis was observed.

The prognostic staging of feline lymphoma based on lesion localization and distribution in I-V stages categorizes all paraspinal or epidural tumors regardless of other tumor sites as stage III, and initial involvement of the CNS and/or bone marrow in stage V (43). Although there is not a consensus about the clinical relevance of lymphoma staging there are indications that it affects prognosis (9). In cats, as in dogs and humans, the wide variation of lymphomas in tumor type and grade would translate to a wide variation in biologic behavior and treatment response (16). There are insufficient studies on differences in the distribution of T, B, and NK cell lymphomas, or low and high-grade lymphomas of the nervous system (20). Lymphoid neoplasms show considerable variations in cellular origin and biological behavior. The present and previous studies (40) illustrate the need for further studies on feline lymphomas that may facilitate classification into anatomical patterns and cytological subtypes. Categorization may be used to develop individual prognostic profiles (9). In humans, diagnostic suspicion of primary CNS lymphoma on MRI may prompt early biopsy rather than conservative management in the appropriate clinical setting (12). Moreover, the severity of necrosis on the histologic specimen on human primary brain lymphoma has been significatively correlated with hyperintense MR signal on T2W images and with rim enhancement (12). Conversely, in our group of cats when this correlation was studied, the presence of hyperintensity on T2W sequences decreased the probability of necrosis. This finding may reflect a difference in the nature between human intracranial primary lymphoma and feline spinal cord lymphomas, but it must be taken into account that in our study biopsy or necropsy were not available for all cats, therefore the presence of tissue necrosis could have been missed in some cases, thus making a bias in the correlation with the MRI features.

## Conclusion

In our group of 27 cats with spinal lymphoma, all of them were evaluated for progressive neurological deficits, commonly acute or subacute, with paraparesis as the most common sign (62.9%), and pain present in 1/3 of the cases. Age at presentation appeared bimodal and related to FeLV status, with 40.7% of cats 2.5 years or younger (63.6% of them FeLV positive), and 44.4% of cats 8 years or older (16.7% of them FeLV positive). In MRI studies spinal lymphomas were most commonly shown as a lumbar/lumbosacral epidural lesion, circumferential to the spinal cord, hyperintense on T2W images, and isointense on T1W images, with variable degrees of post-contrast enhancement and bone involvement. When the T2W signal was not hyperintense it increased the probability of the presence of necrosis in histopathological studies as an unfavorable prognostic factor. The majority of the lesions were focal (81.5%) but tended to extend to >1 vertebral body (66.6%). In the follow-up MRI studies performed in 4 cases new spinal lesions on different localizations and extensions were present in 3 of them. Diagnostic confirmation of spinal lymphoma was performed by CSF, FNA, surgical biopsy, necropsy, or a combination of them. For sample planning, the findings from the MRI studies regarding localization and distribution of the lesion/s were highly valuable. Antemortem diagnosis was achieved in 81.5% of cases. MRI allowed a presumptive diagnosis and had a high concordance with pathological studies regarding lesion localization and extension. The histopathology of the lesions was variable, with most of the tumors graded as intermediate. Lymphoma with spinal involvement in the cat has a guarded prognosis. A fast and accurate identification of the process, facilitated by MRI and confirmed by cytology, may allow better counseling to the owners, with earlier and more precise treatments which may lead to prognosis improvement.

## Limitations

The main limitation of the present report is the retrospective design. Not all the data were available for all the cats and the actual survival times are not known for all the cats as some were lost to follow-up. The imaging studies were performed with different magnets and although the protocols were standardized some differences were present. Histopathology was not available for all patients, and immunophenotyping allowing further classification of specific tumor types was not performed in all cases. Moreover, in the cases with a multifocal pattern, not all the lesions that were observed on MRI were sampled premortem, and the specimens may not fully reflect the histologic nature of all lesions.

Although this case series is the largest at present time, the relatively low number of cases, even including several referral centers, is also a limitation and highlights the difficulty in collecting well-documented cases.

## Data availability statement

The raw data supporting the conclusions of this article will be made available by the authors, without undue reservation.

## Ethics statement

The animal study was reviewed and approved by authors declare that human ethics approval was not needed for this study. Sampling and imaging procedures were held in a clinical environment as part of the diagnostic protocol for medical purposes, with the signed consent of the owners. The material used for the study did not require supplementary procedures or handling. Written informed consent was obtained from the owners for the participation of their animals in this study.

## Author contributions

VL: conception and design of the study, acquisition, analysis and interpretation of data, and drafting and revising the article for intellectual content. JR, MB, and MP: acquisition of data and drafting and revising the article for intellectual content. JM and MM: acquisition of data and revising the article for intellectual content. CB, TL, and AG: acquisition of data. All authors approved the submitted version.

## Conflict of interest

The authors declare that the research was conducted in the absence of any commercial or financial relationships that could be construed as a potential conflict of interest.

## Publisher's note

All claims expressed in this article are solely those of the authors and do not necessarily represent those of their affiliated organizations, or those of the publisher, the editors and the reviewers. Any product that may be evaluated in this article, or claim that may be made by its manufacturer, is not guaranteed or endorsed by the publisher.
